# Model-Based Evaluation of Transmissibility and Intervention Measures for a COVID-19 Outbreak in Xiamen City, China

**DOI:** 10.3389/fpubh.2022.887146

**Published:** 2022-07-13

**Authors:** Weikang Liu, Zhinan Guo, Buasiyamu Abudunaibi, Xue Ouyang, Demeng Wang, Tianlong Yang, Bin Deng, Jiefeng Huang, Benhua Zhao, Yanhua Su, Chenghao Su, Tianmu Chen

**Affiliations:** ^1^State Key Laboratory of Molecular Vaccinology and Molecular Diagnostics, School of Public Health, Xiamen University, Xiamen, China; ^2^Xiamen Center for Disease Control and Prevention, Xiamen, China; ^3^Zhongshan Hospital, Fudan University (Xiamen Branch), Xiamen, China

**Keywords:** COVID-19, dynamics model, transmissibility, intervention, evaluation

## Abstract

**Background:**

In September 2021, there was an outbreak of coronavirus disease 2019 (COVID-19) in Xiamen, China. Various non-pharmacological interventions (NPIs) and pharmacological interventions (PIs) have been implemented to prevent and control the spread of the disease. This study aimed to evaluate the effectiveness of various interventions and to identify priorities for the implementation of prevention and control measures.

**Methods:**

The data of patients with COVID-19 were collected from 8 to 30 September 2021. A Susceptible-Exposed-Infectious-Recovered (SEIR) dynamics model was developed to fit the data and simulate the effectiveness of interventions (medical treatment, isolation, social distancing, masking, and vaccination) under different scenarios. The effective reproductive number (*R*_*eff*_) was used to assess the transmissibility and transmission risk.

**Results:**

A total of 236 cases of COVID-19 were reported in Xiamen. The epidemic curve was divided into three phases (*R*_*eff*_ = 6.8, 1.5, and 0). Notably, the cumulative number of cases was reduced by 99.67% due to the preventive and control measures implemented by the local government. In the effective containment stage, the number of cases could be reduced to 115 by intensifying the implementation of interventions. The total number of cases (*TN*) could be reduced by 29.66–95.34% when patients voluntarily visit fever clinics. When only two or three of these measures are implemented, the simulated *TN* may be greater than the actual number. As four measures were taken simultaneously, the *TN* may be <100, which is 57.63% less than the actual number. The simultaneous implementation of five interventions could rapidly control the transmission and reduce the number of cases to fewer than 25.

**Conclusion:**

With the joint efforts of the government and the public, the outbreak was controlled quickly and effectively. Authorities could promptly cut the transmission chain and control the spread of the disease when patients with fever voluntarily went to the hospital. The ultimate effect of controlling the outbreak through only one intervention was not obvious. The combined community control and mask wearing, along with other interventions, could lead to rapid control of the outbreak and ultimately lower the total number of cases. More importantly, this would mitigate the impact of the outbreak on society and socioeconomics.

## Introduction

Today, the world remains under immense pressure from the coronavirus disease 2019 (COVID-19) pandemic (1). Over the past 2 years, the pandemic has posed an enormous challenge to health systems and a burden on people's health worldwide (2, 3). As of 14 February 2022, the World Health Organization has reported more than 400 million confirmed cases worldwide. Since 2020, China has implemented several non-pharmaceutical interventions (NPIs), such as travel bans, nucleic acid screening, mask wearing, and case isolation to address the spread of the disease, surely, we have indeed achieved good results (4–6). However, there are many constraints in implementing NPIs, for example, it requires a high socioeconomic system and good public cooperation, and it is indisputable that not all countries in the world can achieve the goal of isolation through measures such as lockdown (7, 8). Therefore, vaccine and antiviral therapies, known as pharmacological interventions (PIs), also play an important role in preventing and controlling the spread of COVID-19 (9–11). In several COVID-19 outbreaks in China, the disease spread was effectively controlled by a combination of NPIs and PIs (12, 13).

On 21 September 2021, a confirmed case of COVID-19 was reported in Xiamen City, followed by an outbreak that lasted for a month with a total of 236 infected cases. The outbreak spread to five districts in Xiamen, with the highest number of cases being in the Tong'an District, which was 208 cases. Based on the analysis of the epidemiological characteristics of the epidemic, a transmission dynamic model was used to simulate the development trend in a variety of scenarios, which in turn assessed the effectiveness and priority of different interventions. Moreover, recommendations with public health significance for future epidemic prevention and control programs were made (14, 15). Ever since the COVID-19 pandemic, researchers have conducted a large number of modeling, prediction, and control simulations (16–19). For example, a study explained the cross-age transmission pattern of COVID-19 by constructing a SEIAR model to assess the disease's transmission capacity across age groups (18). Another study simulated the public health impact of the future application of antiviral drugs for the prevention and control of COVID-19 by establishing a transmission dynamics model (17). Moreover, researchers have used models to assess the transmissibility of COVID-19 at different exposure levels by investigating the exposure patterns of the population (19). The above-mentioned studies evaluated the changes brought by single interventions. However, in most real-world cases, outbreaks are controlled by implementing multiple interventions concurrently. Therefore, this study used a model to quantitatively analyze the effects of combinations of multiple interventions and to simultaneously provide optimized solutions for future outbreak management to prevent the uneven distribution of medical resources and reduce the socioeconomic and public health burden of the outbreak.

We developed a Susceptible-Exposed-Infectious-Recovered (SEIR) transmission dynamics model to fit the COVID-19 outbreak data in Xiamen, and simulated the interventions implemented during the outbreak to quantitatively evaluate the effects of various interventions and determine the priorities of prevention and control measures. Our results provide a theoretical basis and an important reference not only for the scientific response and prevention of possible COVID-19 outbreaks but also for optimizing prevention and control programs to reduce the impact of the outbreak on the national economy and public health.

## Methods

### Data Collection

Daily reporting data for COVID-19 from 8 to 30 September 2021 were collected from Xiamen Center for Disease Control and Prevention (https://hfpc.xm.gov.cn/). The demographic data of the population in Xiamen were collected from the seventh national population census in 2020 (https://tjj.xm.gov.cn/tjzl/ndgb/202105/t20210527_2554550.htm).

### Model Development

A SEIR transmission dynamics model without intervention was developed to estimate the transmission capacity and risk of COVID-19 in Xiamen. A Susceptible-Exposed-Infectious-Recovered-Quarantined-Vaccinated (SEIRQV) transmission dynamics model with interventions was used to evaluate the effectiveness of various interventions.

### SEIR Model Without Intervention

Based on our previous study (12–21), we developed a SEIR dynamic model to estimate the spread of COVID-19. The population was divided into Susceptible (S), Exposed (E), Infectious (I), and Recovered/Removed (R) classes.

The model is based on the following assumptions:

(1) Assuming that the infectivity coefficient after effective contact between S and I is β, then at time *t*, the number of newly infected is βSI/*N*.
(2) At time *t*, the number of people who change from E to I is *w*E.
(3) Assuming that the time interval from onset of case I to the first diagnosis is 1/γ, the number of people who switch from I to R at time t is γI.

The equations for the SEIR model are as follows.


$$\begin{array}{lll} \frac{dS}{dt} & = & -\beta SI/N\\ \frac{dE}{dt} & = & \beta SI/N-pwE\\ \frac{dI}{dt} & = & wE-\gamma I-fI\\ \frac{dR}{dt} & = & \gamma I\\ N & = & S+E+I+R \end{array}$$

### Dynamic Model of COVID-19 Transmission With Intervention

On the basis of the non-intervention SEIR model, we further incorporated pharmaceutical interventions (e.g., medical interventions, vaccination) and non-pharmaceutical interventions (e.g., isolation, increasing social distancing, and mask wearing) into the model to construct the SEIQRV model of interventions. The population was divided into Susceptible (S), Exposed (E), Infected (I), Removed/Recovered (R), Isolated (Q), and Vaccinated (V) cases. The definitions of the seven categories are presented in Table 1.

**Table 1:** Variables in the SEIQRV model of interventions.

**Variables**	**Description**	**Unit**
*S*	Susceptible individuals	Individuals
*V*	Vaccinated individuals	Individuals
*E*	Exposed individuals	Individuals
*I*	Infectious individuals	Individuals
*Q*	Isolated individuals	Individuals
*R*	Recovered/Removed individuals	Individuals
*N*	Total number of population	Individuals

**Figure 1:**
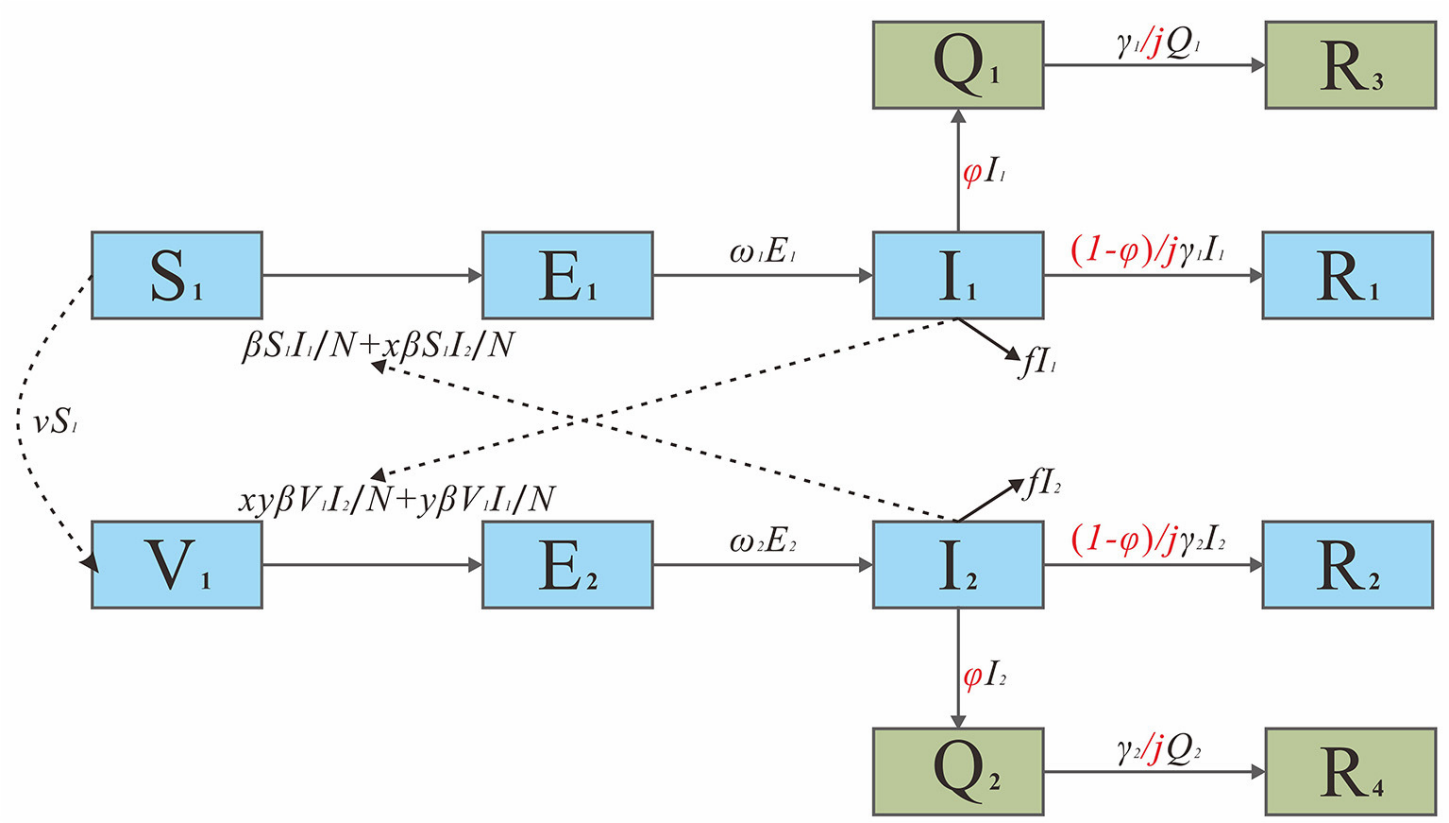
The framework of SEIRQV model of intervention effect evaluation.


The model framework is shown in Figure 1. The equations for the SEIRQV model are as follows.

$$\begin{array}{lll} \frac{dS_{1}}{dt} & = & -\left(\frac{\beta S_{1}I_{1}}{N}+\frac{x\beta S_{1}I_{2}}{N}\right)\\ \frac{dE_{1}}{dt} & = & \left(\frac{\beta S_{1}I_{1}}{N}+\frac{x\beta S_{1}I_{2}}{N}\right)-\omega_{1}E_{1}\\ \frac{dI_{1}}{dt} & = & \omega_{1}E_{1}-\varphi I_{1}-\frac{\left(1-\varphi \right)}{j}\gamma_{1}I_{1}-fI_{1}\\ \frac{dQ_{1}}{dt} & = & \varphi I_{1}-\frac{\gamma_{1}}{j}Q_{1}\\ \frac{dR_{1}}{dt} & = & \frac{\left(1-\varphi \right)}{j}\gamma_{1}I_{1}\\ \frac{dR_{3}}{dt} & = & \frac{\gamma_{1}}{j}Q_{1}\\ \frac{dV_{1}}{dt} & = & -\left(\frac{y\beta V_{1}I_{1}}{N}+\frac{xy\beta V_{1}I_{2}}{N}\right) \end{array}$$

$$\begin{array}{l} \frac{dE_{2}}{dt}=\left(\frac{y\beta V_{1}I_{1}}{N}+\frac{xy\beta V_{1}I_{2}}{N}\right)-\omega_{2}E_{2}\\ \frac{dI_{2}}{dt}=\omega_{2}E_{2}-\frac{\left(1-\varphi \right)}{j}\gamma_{2}I_{2}-\varphi I_{2}-fI_{2}\\ \frac{dR_{2}}{dt}=\frac{\left(1-\varphi \right)}{j}\gamma_{2}I_{2}\\ \frac{dQ_{2}}{dt}=\varphi I_{2}-\frac{\gamma_{2}}{j}Q_{2}\\ \frac{dR_{4}}{dt}=\frac{\gamma_{2}}{j}Q_{2}\\ N=S_{1}+E_{1}+I_{1}+R_{1}+Q_{1}+R_{3}+V_{1}+E_{2}+I_{2}+R_{2}\\ \;+Q_{2}+R_{4} \end{array}$$

In this model, we assumed that medical treatment of an infected person could shorten their infectious period I to *j* times the original one and that the value range of *j* is 0–1. For the scenario of isolation, Q is the number of isolated cases, R_3_ and R_4_ stand for the recovered cases from isolation, and φ is the isolation coefficient. We set δ as the isolation ratio, which could be calculated by δ=R_3_+R_4_/R_1_+R_2_+R_3_+R_4_, we changed the value of δ to simulate the effect of interventions under different isolation ratios. During the isolation period, the disease course was the same as that of those who were not isolated. More importantly, according to existing studies (19), the initial exposure value (*X*) of the current population in China is 15, so the single contact infection rate (*q*):


(1) $$\begin{array}{r} \beta N=1-{\left(1-q\right)}^{X} \end{array}$$

At this point, controlling the source of infection could be seen as increasing the social distance between people by decreasing the contact degree (*X*), while keeping the infectious rate of a single contact constant. The simulation of cutting off transmission also involved measures such as wearing different types of masks or disinfection, to reduce the probability of infection from a single contact. For vaccination measures, we included a vaccinator (V) interval in the model, where *v* represents the vaccine coverage of the population (22). We assumed that *VE* of COVID-19 was similar to that of a previous study (Model 3) (23), which quantified the different protective effects as follows: *VEs* refer to *VE* against susceptibility, *VE*_*I*_ as *VE* against infectiousness. Simultaneously, we included two parameters in the model, where *x* refers to a decrease in the proportion of *VE* for susceptibility and *y* refers to the decrease in the proportion of *VE* for infectiousness. The equations are as follows:


(2) $$\begin{array}{rcl} x & = & 1-VEs \end{array}$$

(3) $$\begin{array}{rcl} y & = & 1-{VE}_{I} \end{array}$$

### Simulation Method

Two scenarios were set to simulate the impact of interventions on the prevention and control of the outbreak in Xiamen. Scenario 1 is a simulation of an integrated intervention, including five sub-scenarios:

Scenario 1 (a): This scenario simulates the situation where in Xiamen failed to promptly test all citizens for nucleic acid or close the community on 13 September, the outbreak continued to spread after 14 September with *R*_*eff*_= 6.88 and after 20 September with *R*_*eff*_= 1.56. Scenario 1 (b): From 13 to 20 September, the transmission capacity of COVID-19 in Xiamen is *R*_*eff*_ = 1.56, at which point the “effective containment phase” of the outbreak occurred. In this scenario, the “effective containment phase” of the outbreak was simulated, and the government intensified its interventions; following which, the transmission capacity would decrease to 4, 1.3, 1.2, 1.1, and 1.

Scenario 1 (c): This scenario simulated the rapid and effective implementation of all prevention and control measures in Xiamen since 13 September. While the transmission capacity within the “effective containment phase” remained the same, it would be shortened from 7 days to 6, 5, 4, 3, 2, 1, and 0 days.

Scenario 1 (d): By assuming that the peak of the outbreak was advanced, this scenario simulated some cases receiving treatment at the fever clinic, to achieve the purpose of “early detection, diagnosis, and isolation,” and to cut off the transmission chain and control the outbreak promptly. Additionally, febrile patients were also simulated to take the initiative to receive medical treatment 1, 2, 3, 4, and 5 days earlier than the peak of the outbreak.

Scenario 1 (e): The “effective containment phase” of the current epidemic would not recur if patients were seen in a timely and proactive manner at fever clinics in designated hospitals, authorities were able to cut the transmission chain in time, and interventions continued to be intensified during the outbreak. The scenario was simulated assuming that the peak of the outbreak was advanced by 1, 2, 3, 4, and 5 days.

In our study, we simulated medical interventions, isolation, social distancing, mask wearing, and vaccination in scenario 2, and evaluated the implementation effects of each of these five interventions, respectively. Meanwhile, we conducted a combination of five interventions, the results of the combination are shown in Table 2, and the effects of each combination of interventions were also evaluated.

**Table 2:** Combination of different interventions.

**Number**	**Types of intervention**	**Mixed intervention**
1	**Mix of two**	Vaccination and medical treatment
2	**interventions**	Vaccination and isolation
3		Vaccination and social distancing
4		Vaccination and wearing mask
5		Medical treatment and isolation
6		Medical treatment and social distancing
7		Medical treatment and wearing mask
8		Isolation and social distancing
9		Isolation and wearing mask
10		Social distancing and wearing mask
11	**Mix of three interventions**	Vaccination and medical treatment and isolation
12		Vaccination and medical treatment and social distancing
13		Vaccination and medical treatment and wearing mask
14		Vaccination and isolation and social distancing
15		Vaccination and isolation and wearing mask
16		Vaccination and social distancing and wearing mask
17		Medical treatment and isolation and social distancing
18		Medical treatment and isolation and wearing mask
19		Medical treatment and social distancing and wearing mask
20		Isolation and social distancing and wearing mask
21	**Mix of four interventions**	Vaccination and medical treatment and social distancing and isolation
22		Vaccination and medical treatment and isolation and wearing mask
23		Vaccination and medical treatment and social distancing and wearing mask
24		Vaccination and isolation and social distancing and wearing mask
25		Medical treatment and isolation and social distancing and wearing mask
26	**Mix of five interventions**	Vaccination and medical treatment and isolation and social distancing and wearing mask

### Parameter Estimation

The SEIR model contains four parameters (β, ω, γ, and *f* ), whose definition, value, and methods of estimation are listed in Table 3. The parameter β can be obtained by fitting the data to the results. According to previous studies (20–23, 25, 26), the value of the parameter ω was set as 0.333 and γ was set as 0.200. By referring to the seventh national census in 2020, the total population was set as 5.28 million, and the case–fatality rate *f* was set as 0%.

**Table 3:** The definition and values of parameters in the SEIR model of COVID-19 in Xiamen City, China (2021).

**Parameter**	**Definition**	**Value**	**Range**	**Source**
β	Transmission relative rate	–	≥0	Curve fitting
*1/*ω	Incubation of symptomatic/asymptomatic	3	3–5	Reference (21)
*1/γ*	Infectious period of symptomatic/asymptomatic	5	5–10	Reference (21)
*f*	Fatality of the disease	0%	0–100%	Actual data
*x*	Decreasing proportion of vaccine efficacy against susceptibility	0.5	0–1	Reference (5, 24)
*y*	Decreasing proportion of vaccine efficacy against infectivity	0.5	0–1	Reference (5, 24)
*1/j*	Medicine treatment effect	4/5, 3/5, 2/5	0~1	Simulate different scenarios
φ	Isolation coefficient	–	0~1	Simulate different scenarios
δ	Isolation ratio	–	0~1	Simulate different scenarios
*X*	Initial exposure value	–	5~15	Simulate different scenarios
*q'*	Single contact infection rate	–	0~100%	Simulate different scenarios

### Estimated Transmission

We have used the effective reproductive number (*R*_*eff*_) to assess the transmissibility and risk of transmission of COVID-19 (27). *R*_*eff*_ is the average number of secondary infections caused by an infected person during one period of infection after the implementation of an intervention. In this study, *R*_*eff*_ is calculated by the definition method. We assume that the natural mortality rate *dr* is 0. The equation of *R*_*eff*_ is as follows:


(4) $$\begin{array}{r} R_{eff}=\frac{\beta S}{\gamma +f+dr} \end{array}$$

In addition to *R*_*eff*_, five other indicators were used to assess transmission capacity and intervention effectiveness, including the total number of new cases (*TN*), total attack rate (*TAR*), peak number of new cases (*NP*), duration of the outbreak (*DO*), and peak time (*PT*). The formulas are as follows:


(5) $$\begin{array}{rcl} TN & = & Total\;number\;of\;new\;cases \end{array}$$

(6) $$\begin{array}{rcl} TAR & = & \frac{TN}{N}\times 100\% \end{array}$$

(7) $$\begin{array}{rcl} DO & = & t_{0}-t_{1} \end{array}$$

(8) $$\begin{array}{rcl} PT & = & t_{P} \end{array}$$

(9) $$\begin{array}{rcl} NP & = & Number\;of\;new\;cases\;at\;peak \end{array}$$

In the above equations, *N*, *t*_0_, *t*_1_, and *t*_p_ refer to the total population, the date of onset of the first case, the date of onset of the last case, and the time of peak onset, respectively.

### Statistics Analysis

Berkeley Madonna, v. 8.3.18 (developed by Robert Macey and George Oster, University of California, Berkeley, CA, United States) was used for parameter fitting and model simulation. The differential equations were solved by the fourth-order Runge Kutta method, and model convergence was based on the least root-mean square (LRMS) method. The fitting degree of the model was evaluated by the determinant coefficient (*R*^2^). SPSS, version 22.0 (IBM Corp, Armonk, NY, United States) was used to perform all statistical analyses, and *p* < 0.05(typically *p*≤ 0.05) was used to indicate statistical significance. GraphPad Prism 7.0 (GraphPad Software, La Jolla, CA, United States) was used to make the charts.

## Results

### Epidemiological Characteristics

**Figure 2:**
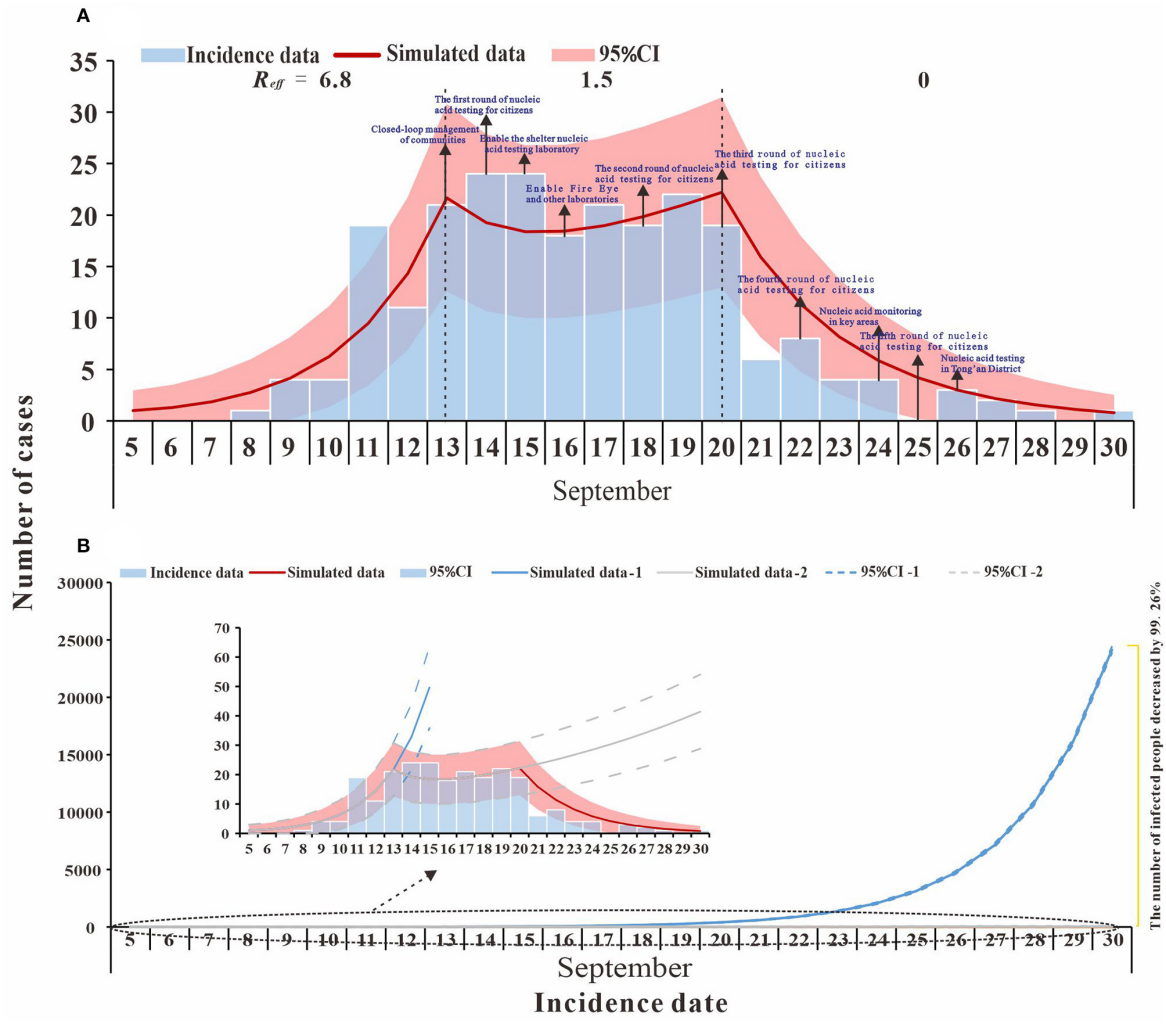
Fitting results of the SEIR model and the data of the actual secondary cases of SARS-CoV-2 infections in Xiamen City, China, 2021. **(A)** evaluation of COVID-19 transmissibility (Reff=6.8, 1.5, and 0); **(B)** effect of intervention measures at different stages.

From 8 to 30 September 2021, a total of 236 cases were reported in Xiamen City, China, with 208 cases reported in the Tong'an District. The first local case was reported on 12 September, with an onset date of 8 September. The last case was reported on 30 September. The peak number of single-day cases was on 14–15 September with the peak number of cases being 24. The epidemiological curve of the local case is roughly divided into three segments: before 13 September, between 13 and 20 September and after 20 September, as shown in Figure 2A.

### Curve Fitting and Transmissibility

The model fitted the outbreak data well (*R*^2^ = 0.837, *p* < 0.001), and we divided the outbreak into three stages, namely, natural transmission, effective containment, and effective control (Figure 2A). The first stage (before 13 September) was the natural transmission, and the effective reproductive number (*R*_*eff*_) for this stage was 6.8. The second stage (from 13 to 20 September) was the effective containment, also known as the “effective containment stage” of the outbreak, in which the effective reproductive number (*R*_*eff*_) was 1.5. The third stage (after 20 September) was the effective control, and the effective reproductive number (*R*_*eff*_) is 0, and all transmission has been effectively blocked.

### Integrated Intervention Simulation

The simulation results for scenario 1 (a) show that Phase I, without intervention, would have resulted in a large outbreak, with an estimated cumulative number of cases and asymptomatic infections of 71,930 as of 30 September. In the second phase, the transmission has been controlled to some extent through contact tracing, nucleic acid screening, and community control. If the transmission continues at *R*_*eff*_= 1.5, the cumulative number of cases and asymptomatic infections is expected to reach 518 by 30 September. In the third phase, local interventions were further strengthened, and the spread of the epidemic (*R*_*eff*_ = 0) was largely interrupted. As of 30 September, there were 236 actual cases and asymptomatic infections, a decrease of 99.67% and 54.44% compared to Phase I and Phase II, respectively (Figure 2B).

**Figure 3:**
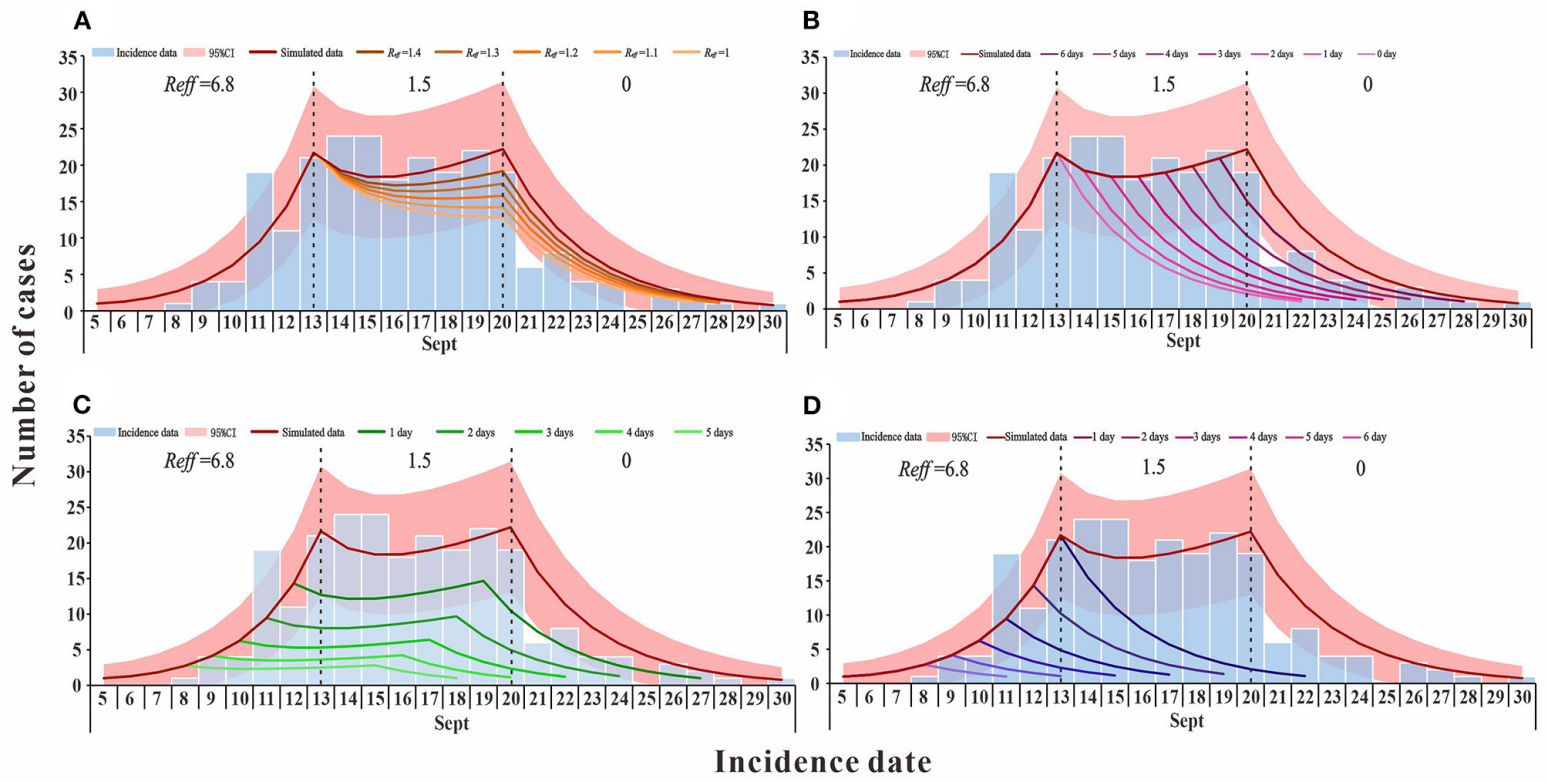
The simulation results of comprehensive intervention measures in Xiamen City, China, 2021. **(A)** simulates the reduction of transmission capacity during the effective containment stage period of the epidemic; **(B)** simulates the decrease in the duration of the epidemic effective containment stage; **(C)** is the scenario simulating the advance of the peak time of the epidemic; and **(D)** simulates the situation where the peak time of the epidemic is advanced and there is no effective containment stage.

In scenario 1 (b), when the outbreak was at the effective containment phase, *R*_*eff*_, which is the transmission capacity, decreased (*R*_*eff*_ = 1.4, 1.3, 1.2, 1.1, and 1), and by 30 September the cumulative number of cases is lower than the actual reported cases (*TN* = 234, 223, 212, 201, and 192) (Figure 3A). If interventions implemented in accordance with scenario 1(c) during the outbreak are timely and effective, they would shorten the duration of the effective containment phase of the outbreak, which would end as early as 22 September, with an expected final cumulative number of cases of 115, a decrease of 51.27% (Figure 3B). The simulation results of scenario 1(d) demonstrate that after febrile patients voluntarily seek treatment, the public health departments can cut the transmission chain in time to advance the peak of the outbreak (1, 2, 3, 4, and 5 days) when the final cumulative number of cases would be 166, 108, 70, 45, and 28, respectively (Figure 3C). In scenario 1 (e), febrile patients have actively sought medical attention and public health authorities have intervened on time to effectively control transmission. The outbreak is no longer in the effective control phase and the final cumulative number of cases would be reduced to <115, with only 11 cases of possible transmission, a 95.34% reduction compared to the actual reported cases (Figure 3D).

### Evaluation of the Effectiveness of Single Interventions

**Figure 4:**
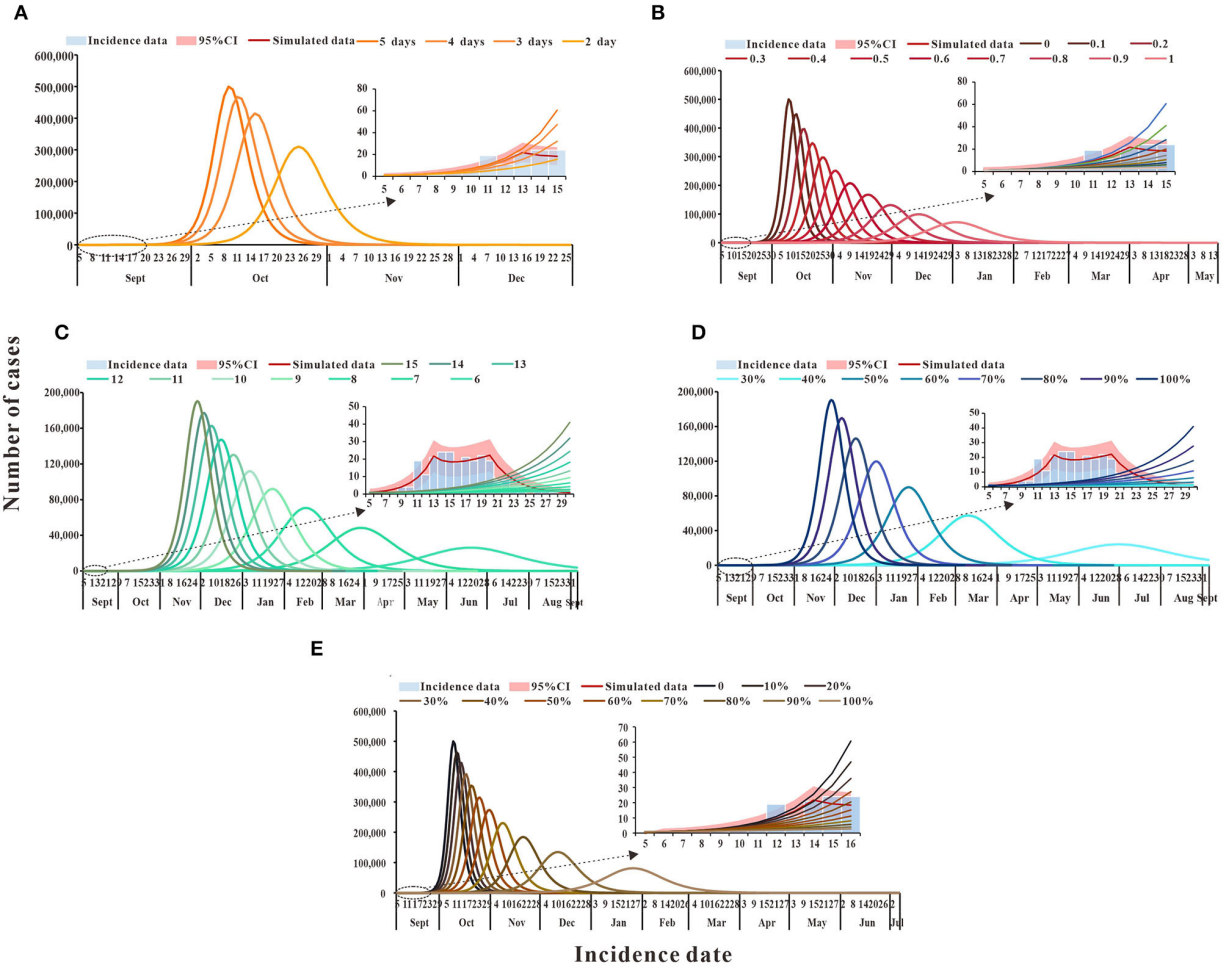
The simulation results of single intervention measures in Xiamen City, China, 2021. **(A)** Medical treatment; **(B)** isolation; **(C)** social distancing; **(D)** wearing masks; and **(E)** vaccination.

According to the simulation results, if only medical treatment interventions were taken after case detection for reducing the infection period (1/γ) from 5 days to 4, 3, or 2 days, then by 30 September, *TN* would be 61,936, 24,395, and 4,515 cases, and *DO* being 88, 93, and 112 days, respectively (Figure 4A). When only the measure of isolation was taken, the cumulative number of cases was more than 2,000 by 30 September because the isolation coefficient was 0.5, then the isolation ratio was 82.25% and, at that point, the *TN* was 1,865. When the isolation coefficient reached 0.9, the isolation ratio was 97.73%, and TN was only 206 (Figure 4B). If social distancing was the only approach taken to reduce the exposure level after the outbreak, the TN would be lower than the actual number of reported cases by 30 September, as the exposure level dropped from the initial 15 to 6, there will be 26 cases when the exposure level is 6 (Figure 4C). When wearing masks is the only way to reduce the probability of transmission (q) for a single exposure and the percentage reduction of q reaches 50%, TN is 40 cases (Figure 4D). When an outbreak occurs, only vaccination could reduce the spread of the virus. By 30 September, TN will be 8,138 cases if the vaccination rate reaches 50% and 732 cases if the vaccination rate reaches 80% (Figure 4E). Specific results for single interventions are shown in Table 4.

**Table 4:** Single intervention effect evaluation.

**Intervention**	**Value**	***TN***	***TAR*(%)**	***NP***	***DO*(days)**	***PT***
Medical treatment	1/γ = 5	5,175,041	99.90%	4,90,910	86	35
	1/γ = 4	5,163,486	99.68%	4,67,276	89	36
	1/γ = 3	5,110,253	98.65%	415,020	94	40
	1/γ = 2	4,839,051	93.42%	310,246	113	50
Isolation	δ = 0	5,175,041	99.90%	499,871	86	34
	δ = 0.1	5,149,841	99.42%	447,934	90	38
	δ = 0.2	5,081,734	98.10%	396,137	99	42
	δ = 0.3	4,962,457	95.80%	347,206	106	46
	δ = 0.4	4,791,643	92.50%	297,301	116	51
	δ = 0.5	4,572,050	88.26%	251,152	130	58
	δ = 0.6	4,307,327	83.15%	207,531	144	65
	δ = 0.7	4,001,156	77.24%	167,242	164	74
	δ = 0.8	3,656,926	70.60%	131,174	185	86
	δ = 0.9	3,277,673	63.28%	99,146	214	100
	δ = 1	2,866,075	55.33%	71,539	253	119
Social distancing	*X* = 15	4,768,827	92.06%	190,351	184	84
	*X* = 14	4,704,152	90.81%	177,153	194	89
	*X* = 13	4,623,350	89.25%	162,698	205	95
	*X* = 12	4,520,942	87.28%	147,174	218	102
	*X* = 11	4,388,981	84.73%	130,245	236	111
	*X* = 10	4,215,618	81.38%	111,847	259	123
	*X* = 9	3,982,609	76.88%	91,933	293	140
	*X* = 8	3,660,666	70.67%	70,638	340	164
	*X* = 7	3,200,409	61.78%	48,336	418	205
	*X* = 6	2,513,171	48.52%	26,135	574	286
Wearing masks	*q'* = 30% *q*	92,445	1.78%	446	1,201	1,201
	*q'* = 40% *q*	2,430,377	46.92%	24,103	598	298
	*q'* = 50% *q*	3,405,601	65.75%	57,343	382	186
	*q'* = 60% *q*	3,956,079	76.37%	89,936	295	142
	*q'* = 70% *q*	4,293,659	82.89%	119,579	249	117
	*q'* = 80% *q*	4,513,411	87.13%	146,096	219	102
	*q'* = 90% *q*	4,663,110	90.02%	169,583	199	92
	*q'* = 100% *q*	4,768,827	92.06%	190,351	184	84
Vaccination	*v* = 0%	5,175,041	99.90%	499,871	86	34
	*v* = 10%	5,157,816	99.57%	464,032	95	36
	*v* = 20%	5,133,295	99.10%	428,638	102	39
	*v* = 30%	5098969	98.44%	391312	109	42
	*v* = 40%	5,051,374	97.52%	353,926	117	45
	*v* = 50%	4,985,540	96.25%	314,707	126	50
	*v* = 60%	4,894,027	94.48%	273,591	139	56
	*v* = 70%	4,763,343	91.96%	230,496	155	64
	*v* = 80%	4,516,211	87.19%	184,199	179	76
	*v* = 90%	2,767,524	53.43%	134,721	220	97
	*v* = 100%	3,833,609	74.01%	81,363	305	143

### Evaluation of the Effect of a Combination of Intervention Measures

**Figure 5:**
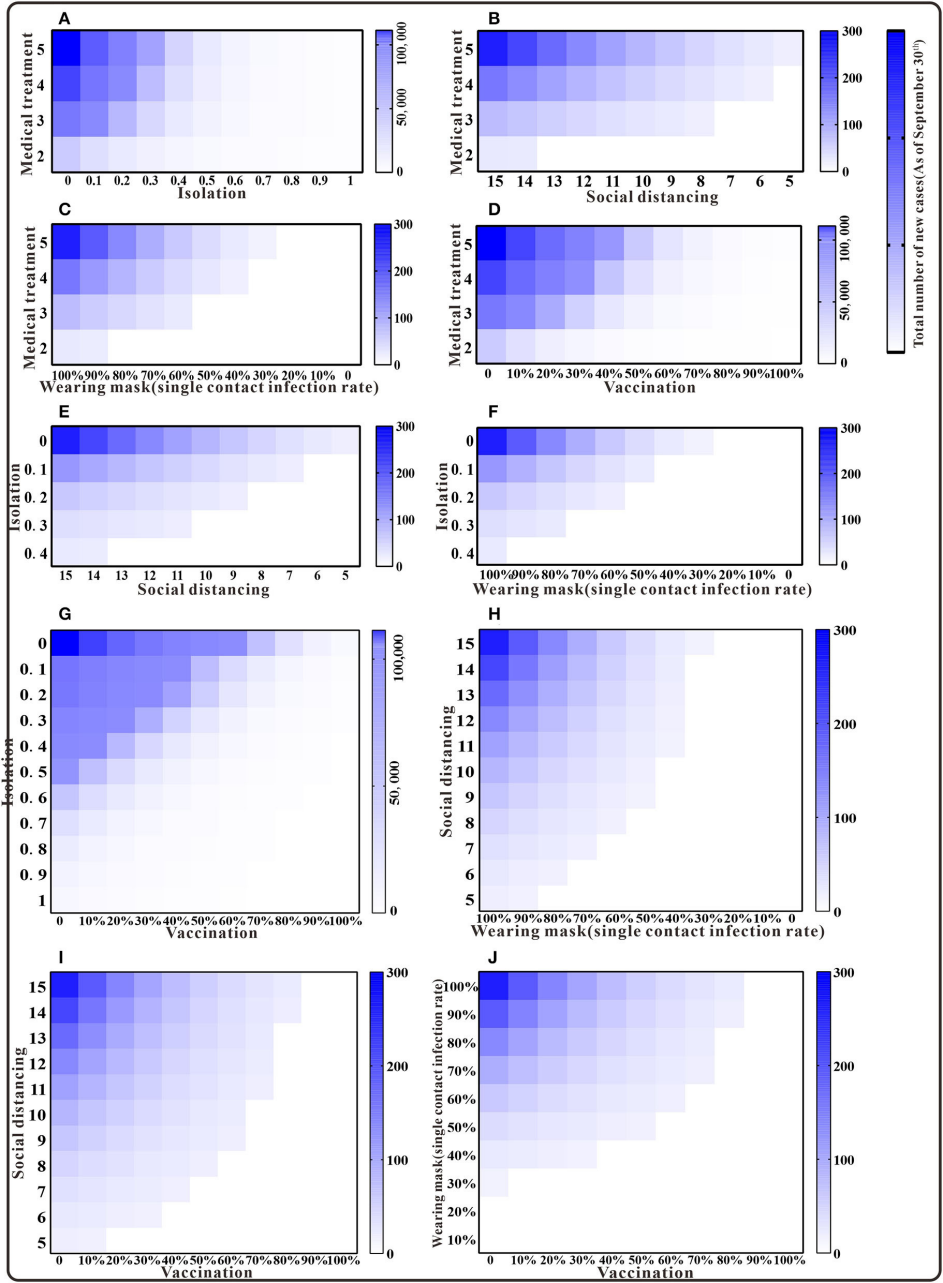
The simulation results of a mix of two interventions in Xiamen City, China, 2021. **(A)** Medical treatment and isolation; **(B)** medical treatment and social distancing; **(C)** medical treatment and wearing mask; **(D)** medical treatment and vaccination; **(E)** isolation and social distancing; **(F)** isolation and wearing mask; **(G)** isolation and vaccination; **(H)** social distancing and wearing mask; **(I)** social distancing and vaccination; and **(J)** wearing mask and vaccination.

Should only two interventions be implemented in combination after case detection, the results of the cumulative number of cases can be found in the Supplementary Table S1. Simulation results demonstrated that medical interventions, combined with social distancing or wearing masks, could keep the *TN* below the actual reported number of cases as of 30 September. When combined with the implementation of quarantine or vaccination, and at a low rate, *TN* would exceed 10,000 cases. That is, the cumulative number of cases can only be effectively reduced under a higher implementation rate. Isolation combined with enhanced social distancing or mask wearing is expected to bring the total number of cases below the reported number by 30 September. The combination of three interventions of enhanced social distancing, mask wearing, and vaccination could reduce *TN* to fewer than 300 cases. When the effects of the interventions are intensified, the cumulative number of cases might be lower than the actual number of cases (Figure 5).

**Figure 6:**
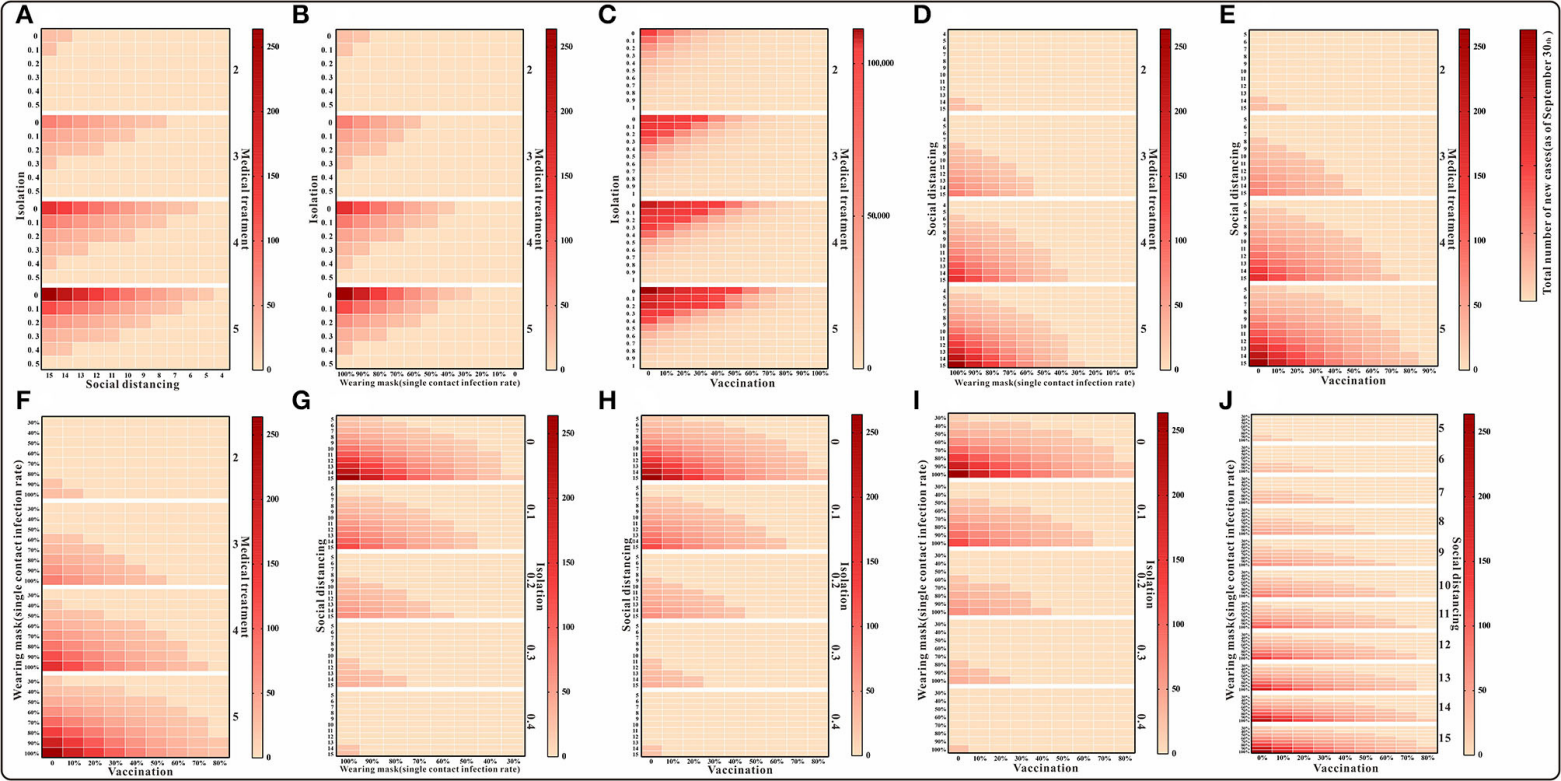
The simulation results of a mix of three interventions in Xiamen City, China, 2021. **(A)** Medical treatment and isolation and social distancing; **(B)** medical treatment and isolation and wearing mask; **(C)** medical treatment and isolation and vaccination; **(D)** medical treatment and social distancing and wearing mask; **(E)** medical treatment and social distancing and vaccination; **(F)** medical treatment and waring mask and vaccination; **(G)** social distancing and isolation and wearing mask; **(H)** social distancing and isolation and vaccination; **(I)** isolation and wearing mask and vaccination; and **(J)** social distancing and wearing mask and vaccination.

Model simulations show that the cumulative number of cases by 30 September was above 10,000 in the case of simultaneous implementation of medical interventions, isolation, and vaccination. When any of the other three interventions were implemented in combination, the cumulative number of cases was fewer than 300. Also, with some enhancement of the intervention, the cumulative number of cases was lower than the actual number of reported cases (Figure 6). The results of the cumulative number of cases can be found in the Supplementary Table S2.

**Figure 7:**
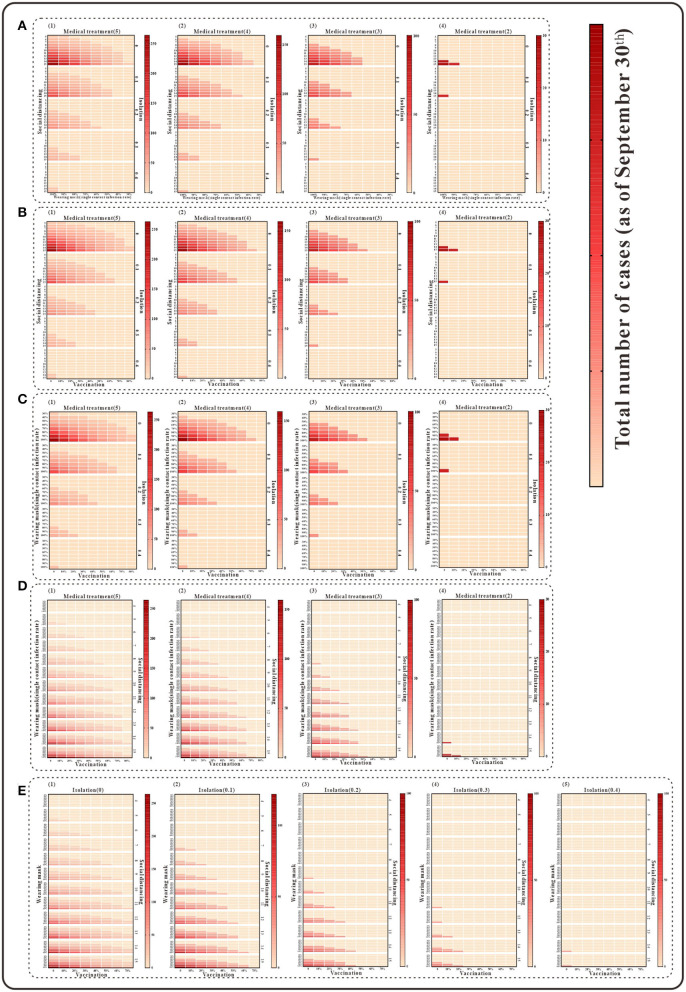
The simulation results of a mix of four interventions in Xiamen City, China, 2021. **(A)** Medical treatment and isolation and social distancing and wearing mask; **(B)** medical treatment and isolation and social distancing and vaccination; **(C)** medical treatment and isolation and wearing mask and vaccination; **(D)** medical treatment and vaccination and social distancing and wearing mask; and **(E)** isolation and vaccination and social distancing and wearing mask.

For the simultaneous implementation of the four measures during the outbreak, two scenarios are discussed. Under the condition that medical intervention reduces the transmission period to <3 days, the simultaneous implementation of any three of the following, namely, isolation, mask wearing, social distancing, and vaccination, the simulation results indicate that the cumulative number of cases as of 30 September would be fewer than 100. If all four measures other than medical intervention are implemented at the same time, the cumulative number of cases as of 30 September will also be <100 when the isolation coefficient is over 0.2 and will continue to decrease as the implementation of the measures enhanced (Figure 7) (detailed in the Supplementary Table S3).

**Figure 8:**
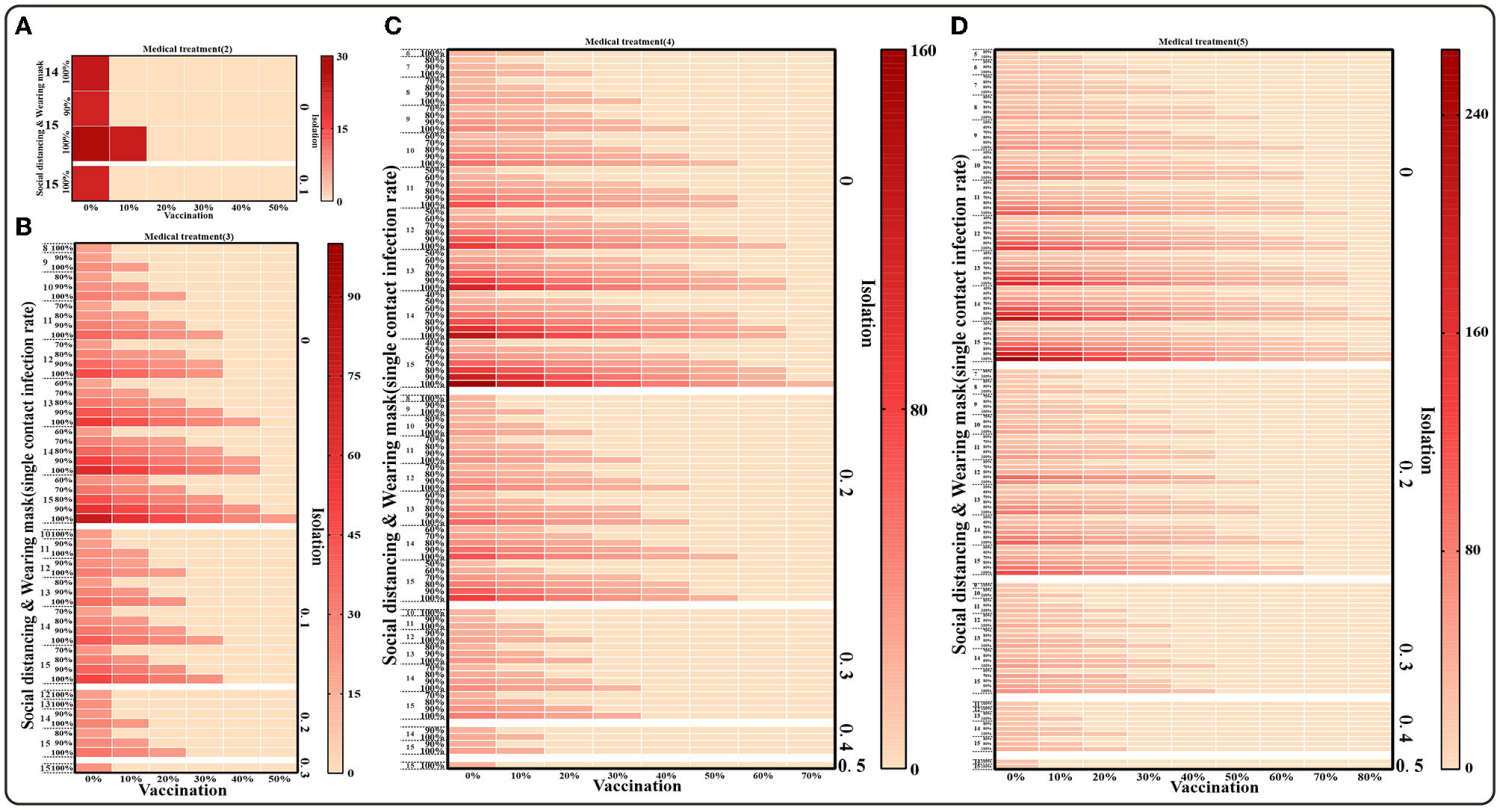
The simulation results of mix of five interventions in Xiamen City, China, 2021. **(A)** Medical treatment (1/γ = 2), Isolation (φ = 0,0.1), Social distancing (*x* = 14,15), Wearing masks (*q* =100%), Vaccination (*v* = 0~50%); **(B)** Medical treatment (1/γ = 3), Isolation (φ = 0–0.3), Social distancing (*x* = 8–15), Wearing masks (*q* = 60%–100%), Vaccination (*v* = 0~50%); **(C)** Medical treatment (1/γ = 4), Isolation (φ = 0–0.4), Social distancing (*x* = 6–15), Wearing masks (*q* = 40%–100%), Vaccination (*v* = 0–70%); **(D)** Medical treatment(1/γ = 5), Isolation (φ = 0–0.5), Social distancing (*x* = 5–15), Wearing masks (*q* = 30%–100%), Vaccination (*v* = 0–80%).

When we assume that the five interventions are implemented simultaneously, as shown in Figure 8, the cumulative number of cases can be reduced to fewer than 150; by adopting medical interventions that reduce the transmission period to two and then implementing the other four interventions in different proportions, the cumulative number of cases can be reduced to fewer than 25 at this point (Figure 8). The results of the cumulative number of cases can be found in the Supplementary Table S4.

## Discussion

In this study, we evaluated the transmission capacity of COVID-19 in Xiamen City in September 2021 by developing a SEIR transmission dynamics model and simulated the effects of implementing various interventions to provide a theoretical basis and effective situation for the implementation of future epidemic prevention and control measures.

From 8 to 30 September 2021, a total of 236 COVID-19 cases were reported in Xiamen City, 208 of which were reported in the Tong'an District. Based on the model fitting results, the spread of this outbreak can be divided into three phases. The *R*_*eff*_ of the first stage is 6.8, which also exceeds the transmission capacity of the Delta variant in previous studies (12, 28), suggesting that there was a breakthrough infection of the Delta variant in this outbreak and that one person can still infect nearly seven people even after the vaccine covered a certain population (29). It is also possible that the outbreak occurred mainly in factories A and B, which were relatively densely populated, resulting in early and rapid transmission of the virus (30). The effective reproductive number in the second stage, *R*_*eff*_ =1.5, indicates that after a series of interventions such as community containment and nucleic acid screening, the transmission of the virus was blocked by 78% and the number of new cases was significantly reduced. However, there was still an 8-day effective containment stage at this time, which prolonged the duration of the outbreak. The effective reproductive number in the third stage (*R*_*eff*_ = 0) indicates that the spread of the disease has been interrupted after effective prevention and control measures by the government and public health authorities.

Also, interventions implemented at different stages of the outbreak were simulated and measures that allowed a rapid and effective containment of the outbreak and their effectiveness were analyzed. The results demonstrated that 71% of the cases occurred during the “effective containment stage” of the outbreak. If public health departments could quickly implement interventions such as community containment and nucleic acid screening during this period, the transmission capacity of the “effective containment stage” of the outbreak would be <1.5, and the outbreak would end 1–3 days earlier. At the same time, the cumulative number of cases will be reduced by 7–18%. If public health authorities can detect cases through nucleic acid screening and isolate the contacts timely during the “effective containment stage” so it lasts no longer than 8 days, the duration of the outbreak will be shortened and the cumulative number of cases would be reduced by 3–51%. Therefore, in the process of epidemic prevention and control, the government and public health departments should pay attention to the effectiveness and timeliness of all interventions, which will not only shorten the duration of the outbreak time or its impact on public life but also reduce the number of cases and the disease burden on people (31, 32).

On the other hand, many cases were found not to have been treated at designated fever clinics, which led to the further spread of the outbreak and put a great threat on the prevention and control of the outbreak. If patients take the initiative to go to hospitals after having symptoms, public health departments can detect the epidemic in time, take prompt measures to cut the transmission chain, and keep the virus from spreading. As shown in the simulation results, proactive consultation of fever cases can advance the peak of the epidemic by 1–5 days, shorten the duration of the epidemic by 3–10 days, and reduce the total number of cases by 29–88%. In the process of active case detection, authorities can take timely and effective interventions to contain it, and if the outbreak does not have an “effective containment phase,” the disease course will be shortened to 4–15 days, the cumulative number of cases will be reduced by 51–95%, and the number of cases will be reduced to 11 after the first onset case is treated promptly (33).

Meanwhile, this study evaluated the effects of medical interventions, isolation, social distance, mask wearing, and vaccination measures through model simulations. The results showed that only one intervention, which is medical intervention, reduced the infection period from 5 days to 2–4 days after the outbreak, when the isolation coefficient reached 0.1–0.9 of cases, increasing social distance reduced people's exposure from an initial value of 15 to 6, and wearing a mask reduced the probability of infection by 0–70% for a single exposure. Increasing vaccination from 0 to 100%, we found that social distancing or wearing a mask had the best effect on reducing the number of cases. In evaluating a combination of 2–4 interventions, it was found that increasing social distance and wearing masks combined with medical interventions, isolation, and vaccination measures would result in a cumulative number of cases of fewer than 200 by the end of the outbreak (34). Thus, effective control can be achieved (8). However, the use of only two of these interventions, that is, medical intervention plus isolation or vaccination, would end up in more than 10,000 cases. Therefore, during the course of an outbreak, public health authorities should take timely community control and homestay surveillance and remind the public of wearing surgical masks correctly, more importantly, ensure that the authorities take care of cases receiving medical treatment while isolating close contacts. Only in this way can the spread of the outbreak and the number of cases be reduced quickly and effectively. Simultaneous implementation of five interventions during an outbreak can reduce the cumulative number of cases to fewer than 150. It was also observed that when medical interventions reduced the transmission period to 2 days, isolation, social distancing, wearing masks, and vaccination were able to reduce the cumulative number of cases to fewer than 25, which allowed the outbreak to be effectively controlled with a minimal impact not only on the public but also on the socioeconomic aspect of the society (35). Although “lockdown of city” or community can effectively increase the social distance between people and prevent the spread of the virus, these measures have a significant impact on socioeconomics and people's daily lives. In this article, we simulated interventions and demonstrated that, on the one hand, in the early stages of an outbreak, symptomatic patients were encouraged to visit sentinel fever clinics, which facilitated early case detection and early outbreak control. On the other hand, in the absence of strict urban containment measures, timely isolation of high-risk populations and the increase in adherence to mask wearing among residents can also prevent the spread of COVID-19 (34). Besides, it is also of great significance that we accelerate drug development, which shortens the infectious period of the outbreak and thus reduces the number of cases (17). The authorities should continue to enhance vaccination strategies for the population to establish an immune barrier to prevent the spread of the virus (36). Consequently, we recommend the early detection and rational management of the outbreak through active consultation and treatment of febrile patients and timely isolation of high-risk populations, as well as continued enhancement of drug and vaccine development to prevent and control possible future COVID-19 outbreaks, ultimately reduce the adverse effects of urban containment and community control measures (37, 38).

### Limitations

This study has some limitations. First, only medical interventions, isolation, social distancing, masking, and vaccine interventions were evaluated in our study, whereas nucleic acid and proximity screening measures implemented at the time of the outbreak were not included in the model simulations. Second, when evaluating vaccination measures, we only considered the population that completed the entire vaccination process and did not consider the effects of different doses. Finally, the natural birth and death rates of the population were not included in the application of the model.

## Conclusions

With the joint efforts of the government and the community, the COVID-19 outbreak in Xiamen has been controlled quickly and effectively. During future outbreaks, isolation measures should be implemented in communities and outbreak sites in time to ensure the social distance between people, thus reducing the level of human contact. Moreover, the public needs to be required to wear masks in a standardized manner, which can rapidly control the spread of the outbreak. Finally, the effectiveness of epidemic prevention and control can be optimized by implementing measures, such as isolation of close contacts, medical treatment of patients, and vaccination.

## Data Availability Statement

The datasets used and analyzed during the current study are available from WL (1320896250@qq.com) on reasonable request.

## Author Contributions

TC, WL, ZG, and CS designed research. WL, ZG, BA, XO, DW, TY, JH, and BD analyzed data. TC, CS, ZG, WL, BA, BD, TY, XO, DW, and JH conducted the research and analyzed the results. TC, CS, WL, ZG, and BA wrote the manuscript. All authors read and approved the final manuscript.

## Funding

This work was partly supported by the Bill and Melinda Gates Foundation (INV-005834) and the National Key Research and Development Program of China (2021YFC2301604).

## Conflict of Interest

The authors declare that the research was conducted in the absence of any commercial or financial relationships that could be construed as a potential conflict of interest.

## Publisher's Note

All claims expressed in this article are solely those of the authors and do not necessarily represent those of their affiliated organizations, or those of the publisher, the editors and the reviewers. Any product that may be evaluated in this article, or claim that may be made by its manufacturer, is not guaranteed or endorsed by the publisher.

## Supplementary Material

Supplementary Table 1 Cumulative number of cases in combination of two interventions (as of 30 September).

Supplementary Table 2 Cumulative number of cases in combination of three interventions (as of 30 September).

Supplementary Table 3 Cumulative number of cases in combination of four interventions (as of 30 September).

Supplementary Table 4 Cumulative number of cases in combination of five interventions (as of 30 September).
